# Hsa_circ_0000301 facilitates the progression of cervical cancer by targeting miR-1228-3p/IRF4 Axis

**DOI:** 10.1186/s12885-021-08331-4

**Published:** 2021-05-21

**Authors:** Zhi-Min Deng, Fang-Fang Dai, Quan Zhou, Yan-Xiang Cheng

**Affiliations:** 1 Department of Obstetrics and Gynecology, Renmin Hospital of Wuhan University, Wuhan, Hubei 430060 P.R. China; 2 Department of Obstetrics and Gynecology, the People’s Hospital of China Three Gorges University/the First People’s Hospital of Yichang, Yichang, Hubei 443000 P.R. China

**Keywords:** Cervical cancer, Bioinformatics, Weighted gene co-expression network analysis, circularRNA-microRNA-mRNA network, IRF4

## Abstract

**Background:**

With the broadened application of gene expression profiles analysis, the role of miRNA and circRNA are of increasing concern in recent years, especially during the pathogenesis of cancer. However, to date, the reported on this area in cervical cancer are limited.

**Method:**

In this study, Weighted Gene Co-Expression Network Analysis (WGCNA) and differential gene expression analysis were utilized to screen out differentially expressed (DE) circular RNAs in cervical cancer, and then we predicted and screened the combined microRNAs (miRNA) and downstream mRNAs to construct circular (circ)RNA-miRNA-mRNA network.

**Result:**

As a result, a regulatory circular (circ)RNA-miRNA-mRNA with 1 circRNA node, 4 miRNA nodes, 135 mRNA nodes were constructed in an attempt to provide novel biomarkers for the pathogenesis of cervical cancer. In addition, enrichment analysis including Gene Ontology (GO) and Kyoto Encyclopedia of Genes and Genomes (KEGG) were performed on mRNAs in the network. After further screening of mRNAs by two online databases of GEPIA2 and RNAyhrid, precise target genes were obtained. Next, we screened out four target genes (*CXCL16*, *IRF4*, *OAS3* and *PTGER3*) by constructing the protein-protein interaction (PPI) network, and mapped them to the initial network to reconstruct the circRNA-miRNA-mRNA network. Notably, the low expression of *IRF4* was demonstrated to be associated with a significantly poorer overall survival in the GEPIA2 database, which was also verified by the immunofluorescence of the sections in Human Protein Atlas (HPA). The upstream miRNA corresponding to *IRF4* is hsa-miR-1228-3p.

**Conclusion:**

From above concern, it can conclude that hsa_circ_0000301/hsa-miR-1228-3p/IRF4 may be involved in the occurrence and development of cervical cancer. However, the specific mechanism should be further studied and confirmed.

**Supplementary Information:**

The online version contains supplementary material available at 10.1186/s12885-021-08331-4.

## Introduction

Cervical cancer is a common gynecological malignant tumor, which seriously threatens women’s health. Its early diagnosis is difficult, leading to a poor prognosis [1]. Therefore, it is necessary to further study the underlying mechanisms of the occurrence and development of cervical cancer, and to find new therapeutic targets. With the increasing popularity of bioinformatics in gene expression profiles analysis, people’s understanding of the genome has gradually improved. Among them, the microRNA (microRNA, miRNA, miR), a class of single-stranded non-coding RNA (ncRNA) molecules encoded by endogenous genes with a length of about 22 nucleotides, is the most intensely studied [2]. They are involved in the regulation of gene expression after transcription. With the deepening of research, scholars discovered that cyclic RNA (circur RNA, circRNA), a special type of ncRNA molecule, has a regulatory effect on miRNA [3]. Due to its closed loop structure and stable expression characteristics, it has obvious advantages in the development and application as new clinical diagnostic markers. CircRNAs act mainly through a competitive endogenous RNA (ceRNA) mechanism, that is, act as the “molecular sponge” of miRNA, competitively bind to miRNA, thereby reducing free miRNA and its interference with transcription, and achieving the purpose of regulating gene expression [4]. More and more researchers have investigated that the abnormal expression of gene products caused by changes in the level of circRNA may be involved in the occurrence and development of cancer [5–7]. However, so far there are limited reports about this area in cervical cancer. The aim of this study is to construct a regulatory circRNA-miRNA-mRNA network to identify significant genes associated with cervical cancer and to further analyze its prognostic significance. Our study may provide a potential basis for clinical diagnosis and treatment of cervical cancer.

**Fig. 1:**
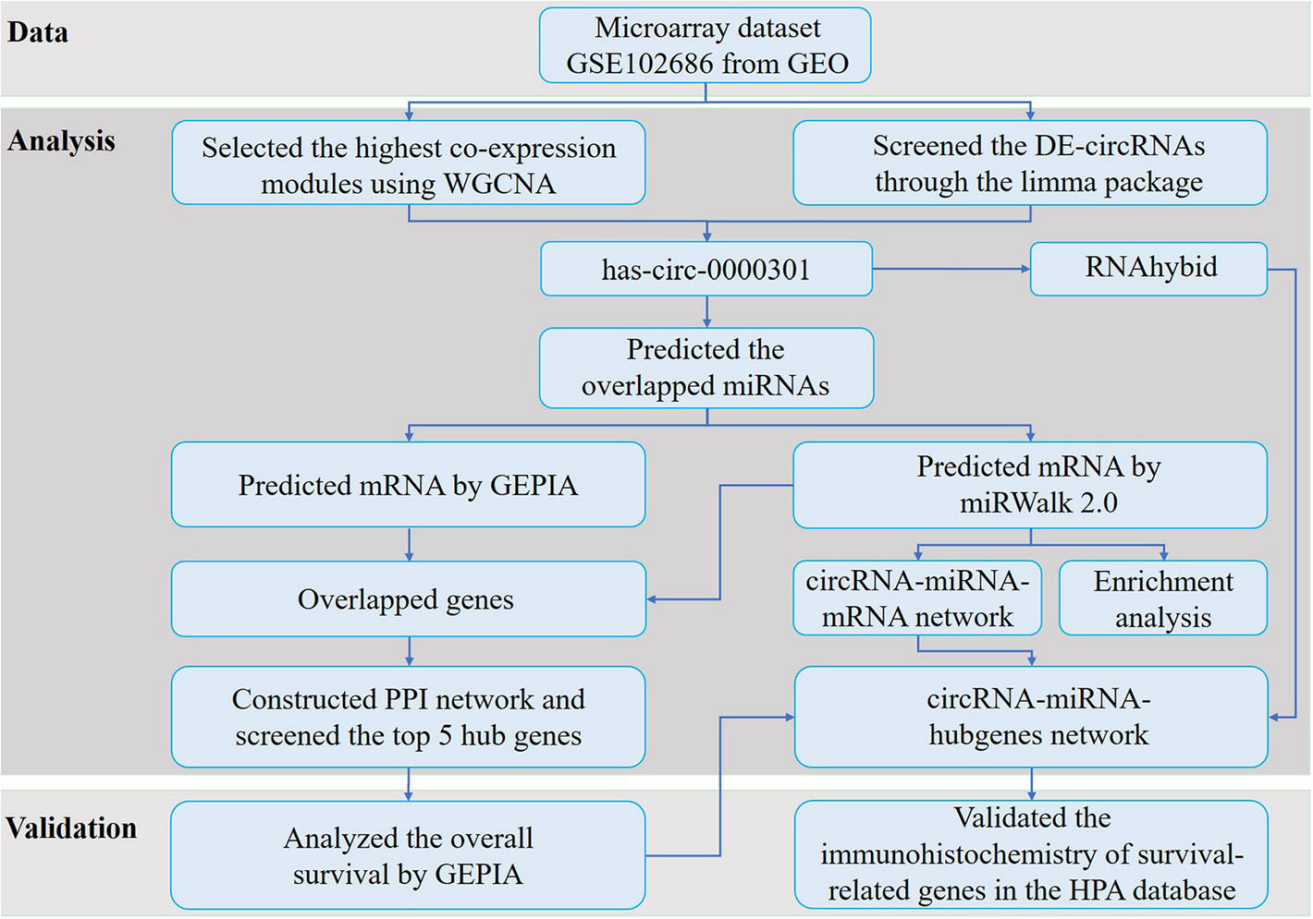
The depicting of the study process. GEO, Gene Expression Omnibus; TCGA, The Cancer Genome Atlas; CESC, cervical squamous cell carcinoma and endocervical adenocarcinoma; DE, differentially expressed; WGCNA, Weighted Gene Co-Expression Network Analysis; GO, Gene Ontology; PPI, protein-protein interaction network; GEPIA, the database of Gene Expression Profiling Interactive Analysis; circRNA, circular RNA; miRNA, microRNA

The workflow of the analysis hub gene extraction curation pipeline is shown in Fig. 1. We elaborate on each step in the following sub-section.

## Materials and methods

### Screening of candidate targeted circRNAs

The normalized expression profiles of GSE102686, a circRNA expression profile chip of cervical cancer from GEO (http://www.ncbi.nlm.nih.gov/geo/), was obtained using *GEOquery* package in R 4.0.2 [8]. GSE102686 consisted of 5 tumor samples and 5 paired normal tissues from patients with cervical cancer, which were studied with the GPL19978 platform. Then, we perform preliminary processing on the data, including converting probe names into gene names, removing duplicate probes, and so on.

In order to screen out circRNAs related to clinical characteristics, WGCNA was employed to perform modular clustering analysis. In our study, the gene co-expression networks were constructed using the *WGCNA* package in R 4.0.2 [9]. In detail, first, the unexpressed and less fluctuating genes based on the expression data were removed. And then, sample clustering and power value calculation were performed to draw a power value scatter plot. In the process of converting the expression matrix to the adjacency matrix, the optimal softpower value was calculated. Based on the Topological Overlap Measure (TOM), after the adjacency matrix was converted into a TOM matrix, the genes were clustered on the basis that the minimum size of each gene network module was set to 50. Moreover, the correlation between each module and the phenotype were calculated according to the feature vector of each module. Modules with high correlation coefficient were considered relevant to clinical features and used for subsequent analysis.

*Limma* package was applied in order to screen out the differentially expressed circRNAs (DE-circRNAs) between cervical cancer and normal tissues in the GSE102686 dataset [10]. Then, the R package *pheatmap* was used to visualize the genes selected based on the cut-off criteria of |logFC| ≥ 1.0 and adj. *p* < 0.05 as a volcano map and a heat map [11]. In order to further reduce the number of DE-circRNA and improve the accuracy of the later prediction, we increased the cut-off criteria of |logFC| to 2. Subsequently, we took intersection of the results from WGCNA and differential gene expression analysis, and Venny 2.1 (https://bioinfogp.cnb.csic.es/tools/venny/index.html) were used to obtain target circRNAs served as candidate biomarkers.

### Construction initial circRNA-miRNA-mRNA network

Following this, the Circbank (http://www.circbank.cn/searchCirc.html) and the Circular RNA Interactome (https://circinteractome.nia.nih.gov/) were jointly applied to predict the binding miRNAs of the target circRNA. Only miRNAs with ≥ 3 binding sites and predicted by these two programs at the same time were regarded as target miRNAs. After that, miRWalk 2.0 (http://zmf.umm.uni-heidelberg.de/apps/zmf/mirwalk2/) [12] was further selected to observe the downstream mRNAs of the collected miRNAs, which included 12 databases. Only those meet the following criteria were selected out as target genes: i) The target gene has overlap in at least 6/12 databases; and ii) the selected 6 databases must include TargetScan and miRanda.

As the final output, we have obtained the core circRNA, the targeted miRNA and the interest genes respectively, and then we can construct the circRNA-miRNA-mRNA network. Thereupon, the network was visualized using Cytoscape software (version 3.7.2) [13].

### Functional annotation for genes of interest and PPI network analysis

Gene Ontology (GO), containing three sub-ontologies—biological process (BP), cellular component (CC), molecular function (MF) [14], and Kyoto Encyclopedia of Genes and Genomes (KEGG) pathway enrichment analyses were performed using Metascape online tool (http://metascape.org/gp/). The differentially expressed genes (DEGs) in the preliminary circRNA-miRNA-mRNA network were identified with a cut-off criterion of *p* < 0.01. The visualization of the enrichment results of CC, MF, and KEGG pathway were provided by Metascape, while BP visualized by the *Goplot* software package in R [15].

In order to narrow the range of target genes, we used GEPIA2 (http://gepia2.cancer-pku.cn/) [16], an online tool for TCGA gene expression and survival analysis, to obtain differentially expressed genes for cervical squamous cell carcinoma and endocervical adenocarcinoma (CESC) with the cut-off criteria of |log_2_FC| ≥ 1.0 and *q*-value < 0.01. Then, the target genes predicted by miRWalk 2.0 and the DEGs of TCGA-CESC obtained on the GEPIA2 website were crossed to obtain more accurate target genes.

Additionally, the STRING (Search Tool for the Retrieval of Interacting Genes) (https://string-db.org/cgi/input.pl) online tool was employed to obtain PPI network for those accurate target genes [17].

### Screening of hub genes and visualizing the miRNA binding sites of the circRNAs

Based on the above-mentioned PPI network, the hub nodes were investigated by the Maximal Clique Centrality (MCC) algorithm in CytoHubba, a plugin in Cytoscape [18]. According to the order of MCC value, the top 5 were defined as hub genes.

In addition, the structure of core circRNAs were identified by the Cancer-Specific CircRNA Database (CSCD) (http://gb.whu.edu.cn/CSCD/) [19]. After that, the RNAhybrid program (https://bibiserv.cebitec.uni-bielefeld.de/rnahybrid) was preformed to predict the miRNA targets of circRNA and their binding methods, and obtain the minimum free energy (MFE) of the duplexes [20].

### Reconstruction of the circRNA-miRNA-hubgene network

Based on the above selection of hub genes and the prediction of circRNA-miRNA binding sites, the preliminary circRNA-miRNA-mRNA network was reconstructed as circRNA-miRNA-hubgenes network.

### Identification and validation of hubgenes

On the one hand, in order to explore the impact of hubgenes on survival, the relationship between hubgenes and Overall Survival (OS) was examined by the GEPIA2. The genes with log-rank *p* < 0.05 was considered statistically significant, that is, the hubgenes were significantly related to the prognosis of cervical cancer.

On the other hand, the expression of prognosis-related hubgenes in tumor tissues and normal tissues were analyzed by Human Protein Atlas (http://www.proteinatlas.org), respectively [21].

## Results

### Construction of weighted gene co-expression modules

**Fig. 2:**
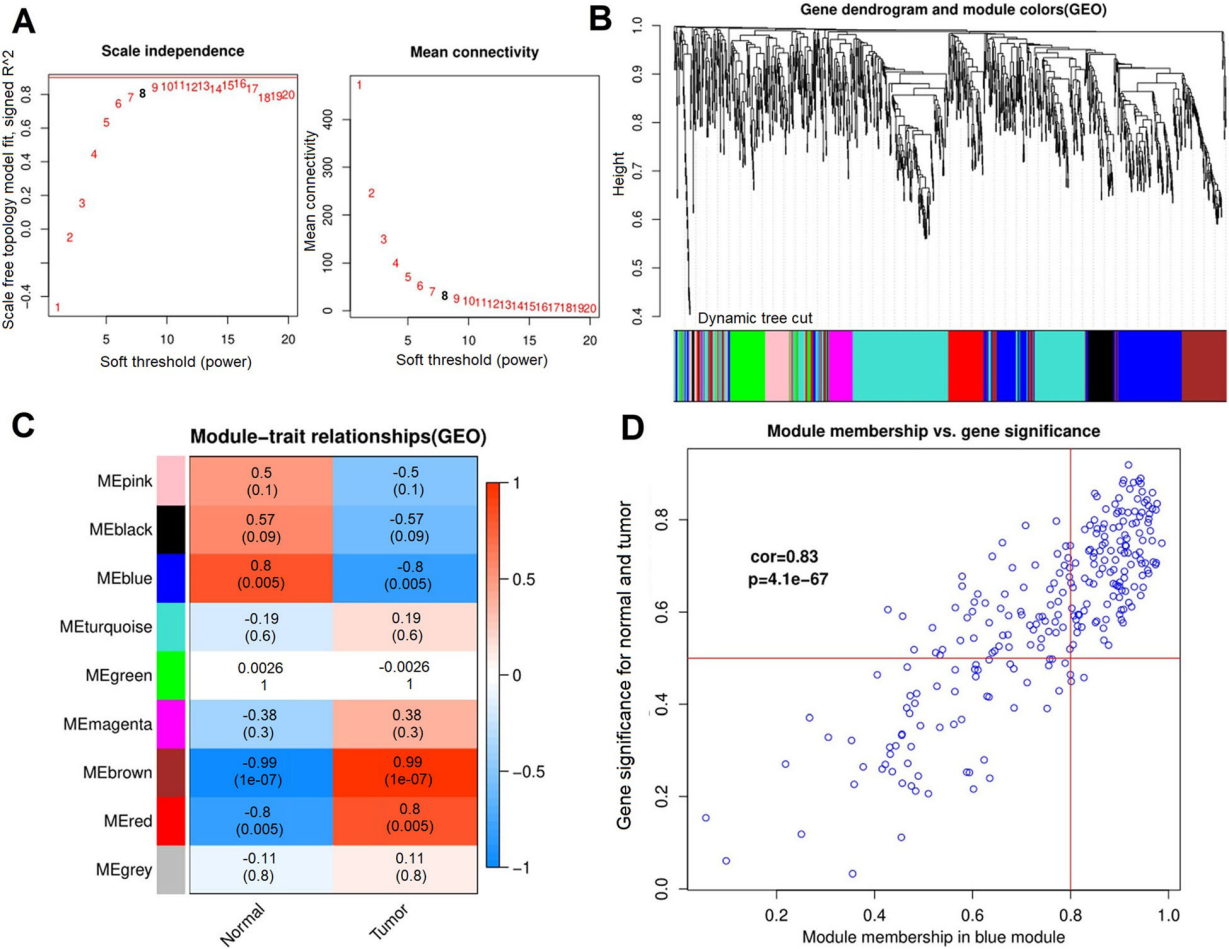
The identification of clinic-associated modules in GSE102686 using the weighted gene co-expression network analysis (WGCNA). **a** Determination of soft-thresholding power. When *β* = 8, R^2^ > 0.8 and mean connectivity < 100, the network was scale-free topology. **b** The Cluster dendrogram of co-expression network modules. **c** Module-trait relationships. Each row corresponds to a color module and each column correlates to a clinical trait (cancer and normal). Each cell contains the corresponding correlation and *p*-value. **d** Scatter plots of the degree and *p*-value of Cox regression in the blue module dataset

In order to find the functional clusters in cervical cancer patients, the gene co-expression networks were constructed from the GSE102686 datasets with the *WGCNA* package. Generally speaking, when the square of the correlation coefficient (*R*^*2*^) is higher, and when the mean connectivity of all genes is less than 100, it means that the network we construct has the characteristics of scale-free topology. It can be seen from Fig. 2a that when the soft threshold is 8, *R*^*2*^ > 0.8 and mean connectivity < 100, indicating that the WGCNA gene network obeys a scale-free distribution at this time. After cluster analysis, a tree diagram was drawn, and a total of 9 modules were identified as shown in Fig. 2b. The results of the module-trait relationships are presented in Fig. 2c, revealing that the brown module possesses the highest correlation with the tissue type. Obviously, the blue module ranks second (brown module: *r* = 0.99, *p* = 1e− 07; blue module: *r* = 0.8, *p* = 0.005). In this study, we selected the blue modules that are positively correlated with normal tissues and negatively correlated with tumor tissues. Figure 2d shows the degree of Cox regression of the blue module data set and the scatter plot of the *p* value. From the figure, it can be seen that the cox value is 0.83, which displays the high correlation.

### Identification DE-circRNA from GEO database and WGCNA co-expression module

**Fig. 3:**
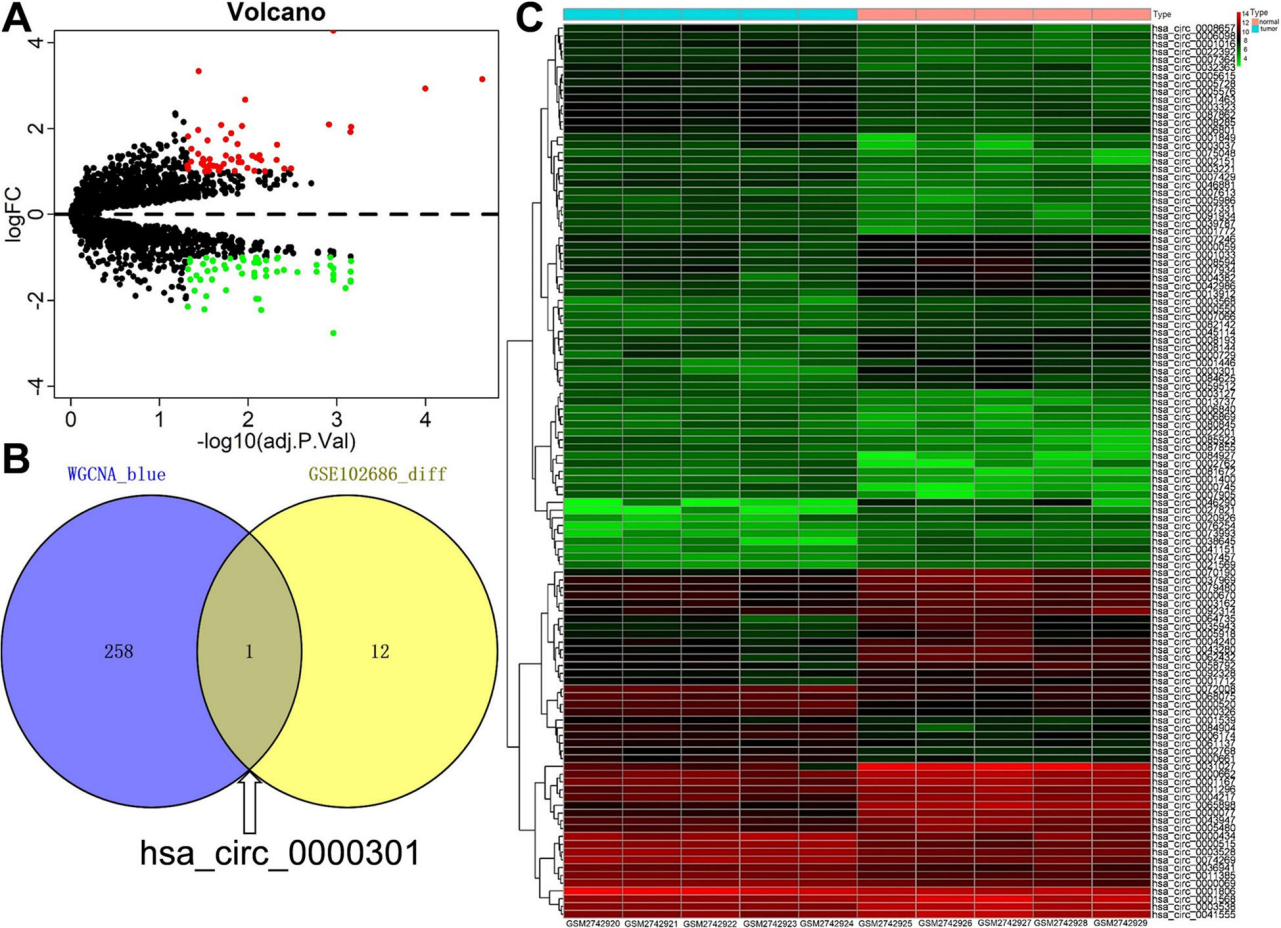
Identification of differentially expressed circRNAs among the GSE102686 dataset of cervical cancer with the cut-off criteria of |logFC| ≥ 1.0 and adj. *p* < 0.05. **a** Volcano plot of DE-circRNAs in the GSE102686 dataset. **b** Heat map of DE-circRNAs in the GSE102686 dataset. **c** The Venn diagram of genes among DE-circRNAs list and co-expression module, when the cut-off criteria of |logFC| is increased to 2. In total, only 1 overlapping circRNA in the intersection of DE-circRNAs list and the co-expression blue module

Based on the cut-off criteria of |logFC| ≥ 1.0 and adj. *p* < 0.05, a total of 115 DE-circRNAs in the GSE102686 dataset was found to be dysregulated in tumor tissues by the *limma* package (Fig. 3a). Next, heat maps were drawn from these 115 DE-circRNAs, and the results showed that compared with normal tissues, 56 circRNAs were up-regulated in cervical cancer, while the other 59 were down-regulated (Fig. 3b). After increasing the cut-off criteria of |logFC| to 2, the number of DE-circRNAs dropped from 115 to 13 (Supplemental Table S1). At last, the 13 DE-circRNAs and the 259 circRNAs contained in the blue module in WGCNA are intersected to obtain the core circRNAs. From Fig. 3c, we can see that only 1 overlapping circRNA in the intersection of DE-circRNAs list and the co-expression blue module, which is hsa_circ_0000301.

### Preliminary circRNA-miRNA-mRNA network

**Fig. 4:**
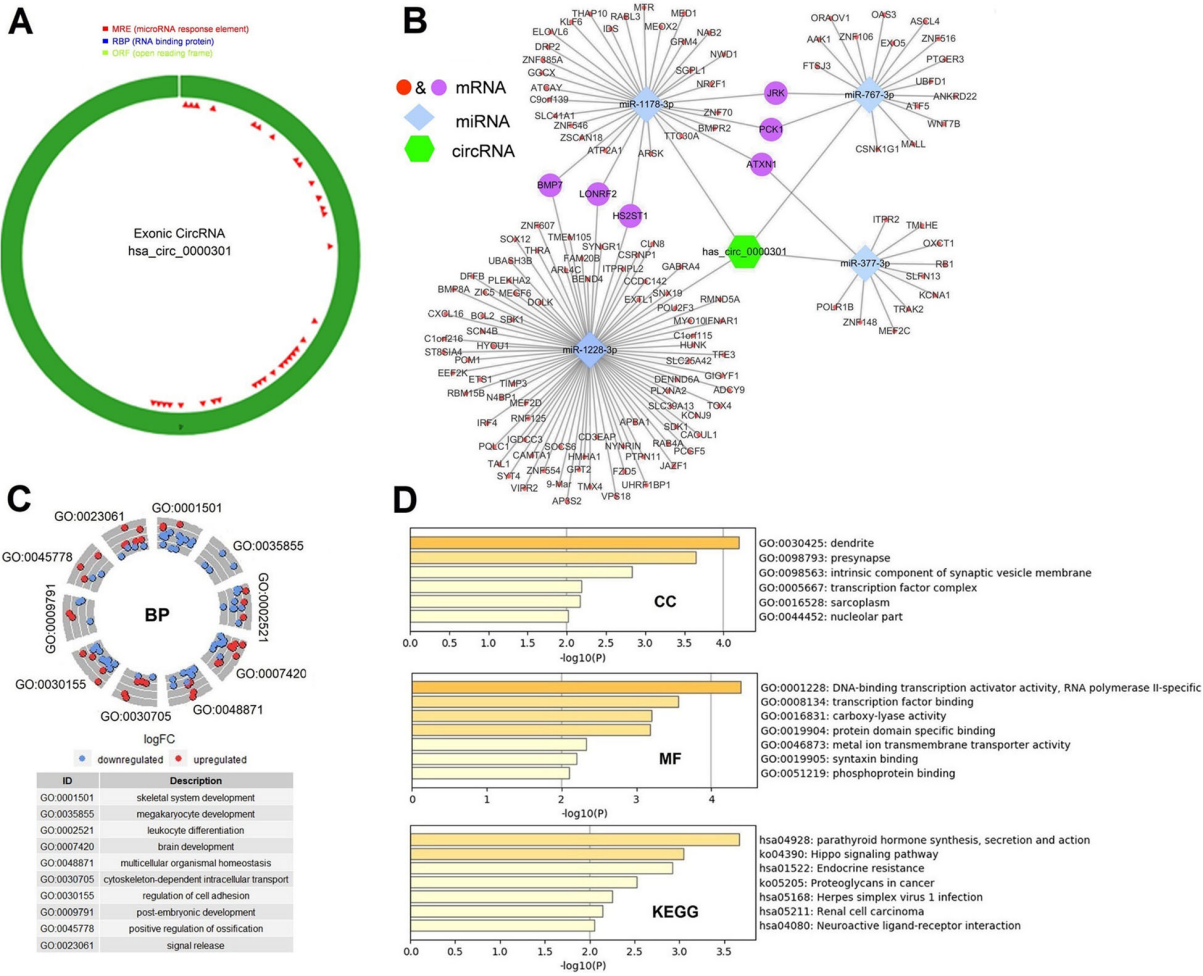
Construction of preliminary circRNA-miRNA-mRNA network. **a** The structure of hsa_circ_0000301. **b** Preliminary circRNA-miRNA-mRNA network was constructed. The network was composed of 1 circRNA node, 4 miRNA nodes, and 135 mRNA nodes. The network presented an initial perception of the association between the hsa_circ_0000301 the 4 miRNAs (hsa-miR-1178-3p, hsa-miR-1228-3p, hsa-miR-377-3p and hsa-miR-767-3p) and the 135 mRNAs. **c** The enrichment results of BP drawn by R software. **d** Enrichment visualization of CC, MF, KEGG pathways

The structure and primary characteristics of hsa_circ_0000301 are demonstrated in Fig. 4a and Table 1, respectively. Subsequently, online website tools Circbank and Circular RNA Interactome programs were applied to predict miRNAs that can bind to hsa_circ_0000301. A total of 4 miRNAs were predicted by these two tools at the same time, and the binding sites were ≥ 3. These four miRNAs are considered to be effective and included in further research (Table 2). Based on this, the target mRNAs of these four miRNAs were predicted in miRWalk 2.0. After removing the duplicates and invalid values, a total of 135 effective miRNAs without duplicates are obtained. Specifically, 32 mRNAs of hsa-miR-1178-3p, 81 mRNAs of hsa-miR-1228-3p, 11 mRNAs of hsa-miR-377-3p, and 17 mRNAs of hsa-miR-767-3p were obtained. In summary, as indicated in Fig. 4b, a preliminary circRNA-miRNA-mRNA network, which contains 1 circRNA node (hsa_circ_0000301), 4 miRNA nodes (hsa-miR-1178-3p, hsa-miR-1228-3p, hsa-miR-377-3p and hsa-miR-767-3p), 135 mRNA nodes are constructed.

**Table 1:** Primary characteristics of has_circ_0000301

circRNA	Chromosome	Start position	End position	Strand	Gene symbol
has_circ_0000301	Chr11	47,379,617	47,379,952	–	SPL1

*circRNA*, circular RNA

**Table 2:** MiRNAs that can bind to has_circ_0000301 are predicted by Circbank and Circular RNA Interactome

MiRNA	Sites
has-miR-1178-3p	6
hsa-miR-1228-3p	6
hsa-miR-377-3p	11
hsa-miR-767-3p	3

*MiRNA*, microRNA; *Sites*, number of binding sites (miRNAs are considered to be effective only when sites ≥ 3)

### Functional annotation for genes of interest

To gain further insight into the potential functions of the 135 mRNAs that obtained by merging the targeted mRNAs of 4 predicted-miRNAs, gene enrichment analysis was performed by the *clusterProfiler* package [22] and Metascape online tool. Figure 4c shows the enrichment results of BP, which are mainly enriched in cell functions, including cell development, differentiation, and adhesion. The enrichment of CC, MF, and KEGG pathways demonstrated the target genes are mainly related to the Hippo signaling pathway, the proteoglycan metabolism pathway in cancer, and neuroactive ligand-receptor interaction (Fig. 4d).

### Reconstruction of the circRNA-miRNA-mRNA network

**Fig. 5:**
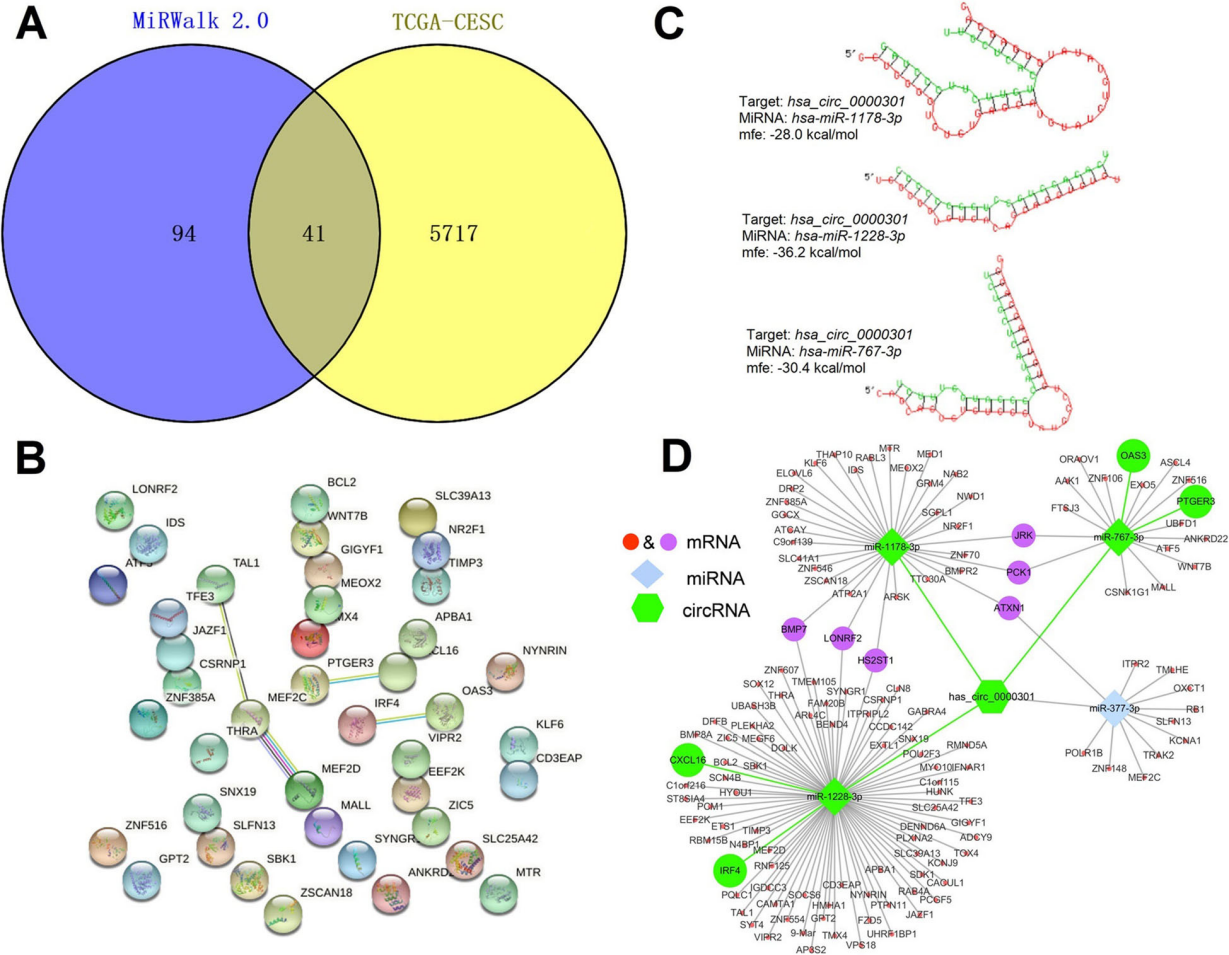
Reconstruction of the circRNA-miRNA-mRNA network. **a** Venn diagram of DE-mRNAs in TCGA-CESC and mRNAs predicted by miRWalk 2.0. **b** PPI network obtained on STRING online website. **c** Binding site between hsa-miR-1178-3p, has-miR-1228-3p, hsa-miR-767-3p, and hsa_circ_0000301 with maximum MFE. **d** The reconstructed circRNA-miRNA-mRNA network

In order to precise the mRNAs in the circRNA-miRNA-mRNA network, 5758 DE-miRNAs in TCGA-CESC were obtained from GEPIA2 and intersected with the 135 mRNAs in the network. Finally, 41 overlapping mRNAs were obtained (Fig. 5a and Supplemental Table S2). And then, the PPI protein mutual aid network is obtained (Fig. 5b). The top 5 hubgenes (*MEF2C*, *CXCL16*, *OAS3*, *IRF4*, *PTGER3*) were calculate via cytoscape. Additionally, the binding site of hsa-miR-1178-3p, has-miR-1228-3p, hsa-miR-767-3p, and hsa_circ_0000301 are indicated. Figure 5c shows the binding site at the maximum MFE of the duplexes in each binding mode and the complete binding modes can be seen in Supplemental Fig. S1. Since the binding sites and binding modes between hsa-miR-377-3p and hsa_circ_0000301 have not been obtained, the target genes of it are not investigated in subsequent studies, including *MEF2C*. The identified hubgenes and miRNAs with binding sites were mapped into the initial network to obtain a reconstructed network, which contains 4 circRNA-miRNA-mRNA regulatory axes (Fig. 5d and Table 3).

**Table 3:** Regulatory axes identified from the circRNA-miRNA-hubgene network

Regulatory axis	circRNA	miRNA	Hubgene
1	hsa_circ_0000301	hsa-miR-1228-3p	CXCL16
2	hsa_circ_0000301	hsa-miR-1228-3p	IRF4
3	hsa_circ_0000301	hsa-miR-767-3p	OAS3
4	hsa_circ_0000301	hsa-miR-767-3p	PTGER3

### Evaluation of overall survival (OS) and the expression of IRF4

**Fig. 6:**
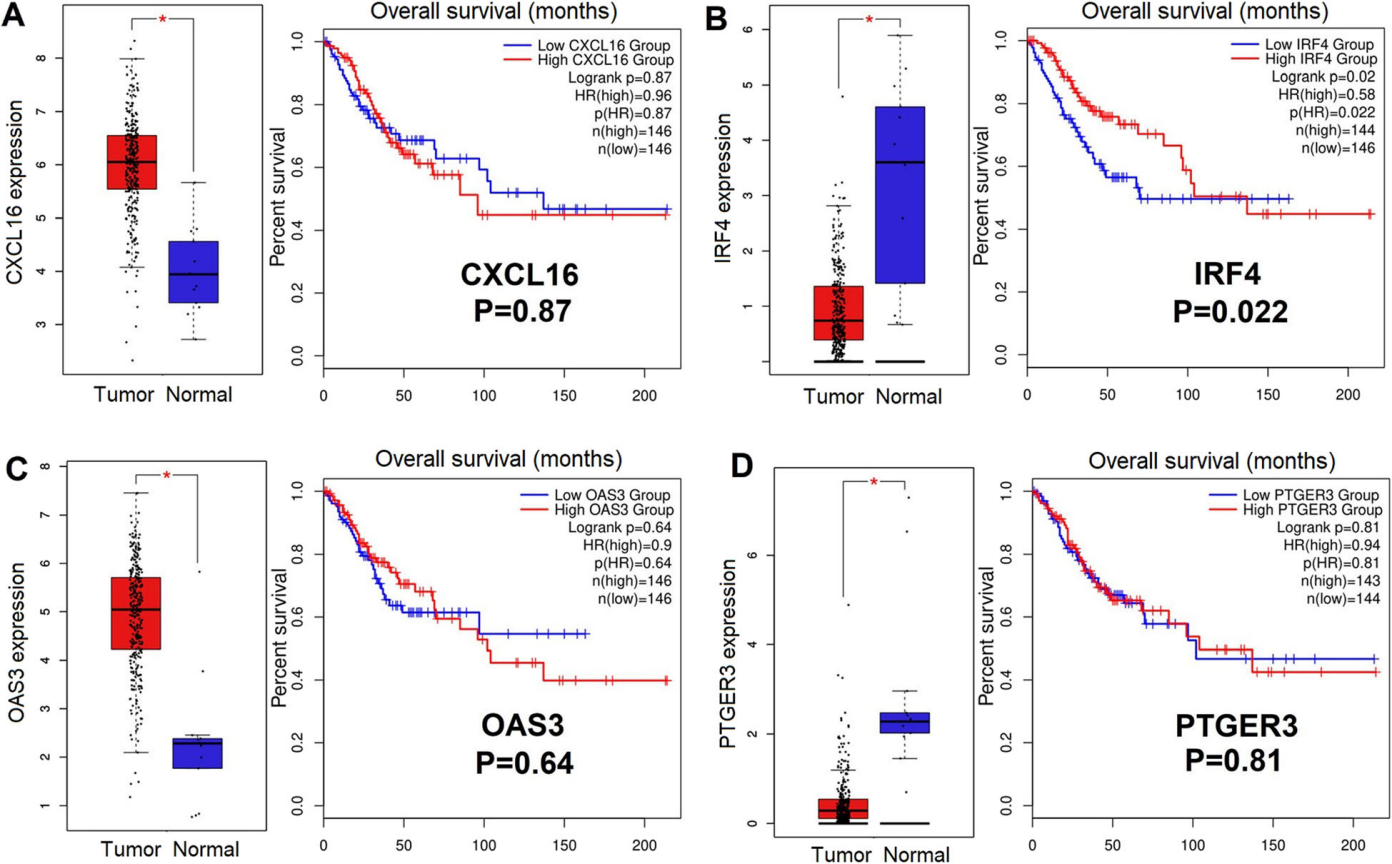
Expression and survival curve of hub genes in CESC and normal tissues based on GEPIA. **a** CXCL16; **b** IRF4; **c** OAS3; **d** PTGER3. Notes: Expression of the hub genes were detected in 306 CESC tissues (T) and 13 normal tissues (N) on the basis of GEPIA

**Fig. 7:**
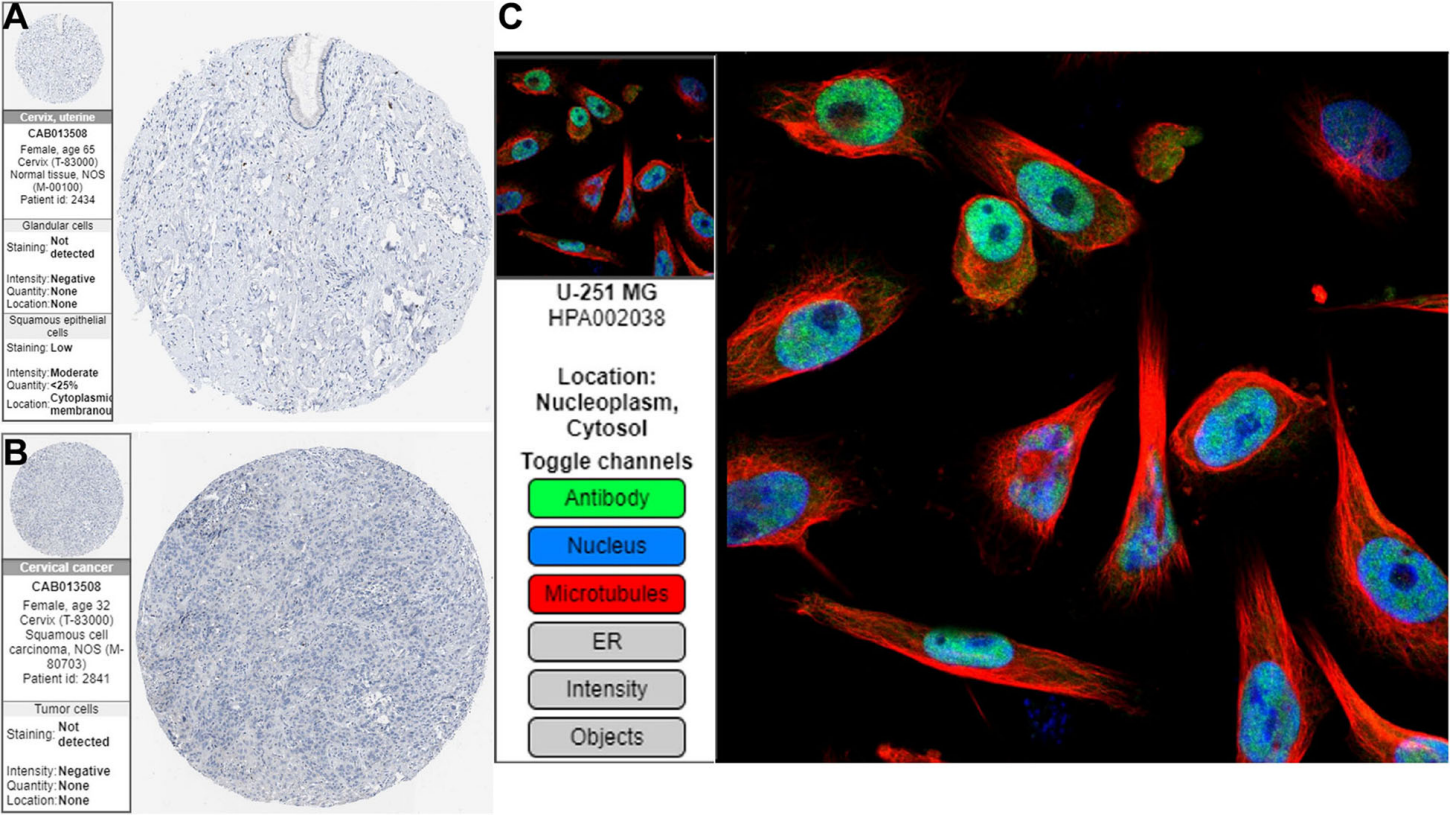
Immunohistochemistry of *IRF4* based on the Human Protein Atlas. **a** The expression of *IRF4* in normal tissue sections. **b** The expression of *IRF4* in cervical cancer tissue sections. **c** The expression position of *IRF4* in the cell

The OS for the 4 hubgenes assess from the GEPIA2 are displayed in Fig. 6a-d. Notably, lower expression of *IRF4* revealed a significantly poorer OS (*p* = 0.022). However, no significant effect was indicated for the remaining 3 hubgenes regarding OS. Therefore, *IRF4* was considered to be hub genes. Figure 7a-b display the immunohistochemical images of *IRF4* in normal tissues and cervical cancer tissues based on the HPA database. It can be seen from the legend that in normal squamous epithelial cells, the staining level of *IRF4* is low, the intensity is moderate, and the quantity < 25%. While in tumor cells, the staining level isn’t detected, the intensity is negative, and the quantity is none, which reveals that *IRF4* expression is down-regulated in tumor tissues. Immunofluorescence staining of *IRF4* in cells is mainly expressed in the nucleoplasm and a small part in the cytosol (Fig. 7c).

## Discussion

Thanks to the rapid development of bioinformatics technology and the improvement of people’s understanding of genomics, the molecular mechanism of cervical cancer has made great progress in the past ten years. The rise of non-coding RNA research has gradually clarified the complex post-transcriptional regulatory network. CircRNA, a new type of non-coding RNA, possesses specific biological functions due to its loop closure. CircRNA acts as a molecular sponge of miRNA, which competitively binds miRNA to regulate gene expression. At present, circRNA mainly regulates the development of cancer through the ceRNA mechanism [23], and there is still a large number of cervical cancer-related circRNAs that need to be explored. Therefore, by analyzing the circRNA expression profile data of cervical cancer, this study constructed a circRNA-miRNA-mRNA network and performed functional enrichment analysis, aiming to screen out potential ncRNAs and target genes involved in regulating the development of cervical cancer. During the construction of the circRNA-miRNA-mRNA network, the targeting relationship of miRNAs-mRNAs were determined by miRWalk 2.0, while the targeting relationship between circRNAs-miRNAs were identified through two online tools (CircBank and Circular RNA Interactome). Subsequently, two methods were chosen to improve the accuracy of mRNA prediction in the initial network: i) Taking the intersection of the DE-mRNAs from the TCGA database and the mRNAs predicted by miRWalk2.0; ii) Eliminating the miRNA whose gene sequence has no binding site with hsa_circ_0000301 by RNAhybrid tools, which based on the MFE evaluation. The above measures ensure the reliability of this signal axis. Besides, enrichment analysis results demonstrated that these mRNAs are mainly involved in the cell differentiation and cell adhesion, Hippo signaling pathway, proteoglycans in cancer, and neuroactive ligand-receptor interaction.

Meanwhile, according to MCC scores from the CytoHubba plugin in Cytoscape, the top 5 cervical cancer-related genes were screened out (*MEF2C*, *CXCL16*, *IRF4*, *OAS3*, *PTGER3*). Among them, low expression of *IRF4* is associated with poor prognosis of cervical cancer. *IRF4*, interferon regulatory factor 4, belongs to the IRF family of transcription factors and is a critical transcriptional regulator of immune system development and function [24]. Expression of *IRF4* can be detected in T, B, DC and macrophages, which is strongly induced by antigen receptor signaling. The current disease researched on *IRF4* mostly focuses on multiple myeloma, lymphoma, and various subtypes of leukemia [25–27]. Studies have also shown that *IRF4* directs Treg differentiation and immunosuppression in human cancers, and IRF4^+^ Tregs are associated with poor prognosis [28]. However, there is almost no research on cervical cancer. As a transcription factor involved in the differentiation of immune cells, *IRF4* may be a good entry point for studying the immune cells and tumor microenvironment of cervical cancer. These results indicate that the hsa_circ_0000301/hsa-miR-1228-3p/IRF4 signaling pathway is likely to function a critical role in the occurrence and development of cervical cancer.

In short, this study analyzed the circRNA expression profile of cervical cancer, predicted the downstream miRNA and mRNA of circRNA via bioinformatics methods. Finally, hsa_circ_0000301/hsa-miR-1228-3p/IRF4 was obtained as a possible regulatory pathway. Our investigation can provide new insights into the pathogenesis of cervical cancer and facilitate to explore new targets for treatment.

## Supplementary Information

**Additional file 1: Supplemental Fig. S1.** The complete binding site between miRNAs and hsa_circ_0000301. (A) hsa-miR-1178-3p & hsa_circ_0000301; (B) has-miR-1228-3p & hsa_circ_0000301; (C) hsa-miR-767-3p & hsa_circ_0000301. **Supplemental Table S1.** Features of the DE-circRNAs in GSE102686 dataset with the cut-off criteria of |logFC| ≥ 2.0 and adj. *p* < 0.05. **Supplemental Table S2.** The target genes of hsa_circ_0000301 in cervical cancer with GEPIA2 and MiRWalk.

## Data Availability

The datasets generated and/or analysed during the current study are available in the GEO repository (https://www.ncbi.nlm.nih.gov/geo/query/acc.cgi?acc=GSE102686).
