# Supplementary material for: Hsa_circ_0000301 facilitates the progression of cervical cancer by targeting miR-1228-3p/IRF4 Axis
Source: BMC Cancer. 2021 May 21;21:583. doi: 10.1186/s12885-021-08331-4 (PMC8140416; doi:10.1186/s12885-021-08331-4)
Supplement: Supplementary file 1 — Additional file 1: Supplemental Fig. S1. The complete binding site between miRNAs and hsa_circ_0000301. (A) hsa-miR-1178-3p & hsa_circ_0000301; (B) has-miR-1228-3p & hsa_circ_0000301; (C) hsa-miR-767-3p & hsa_circ_0000301. Supplemental Table S1. Features of the DE-circRNAs in GSE102686 dataset with the cut-off criteria of |logFC| ≥ 2.0 and adj. p < 0.05. Supplemental Table S2. The target genes of hsa_circ_0000301 in cervical cancer with GEPIA2 and MiRWalk. [file 12885_2021_8331_MOESM1_ESM.pdf]

Hsa_circ_0000301 facilitates the progression of cervical cancer by targeting miR-1228-3p/IRF4 Axis

Zhi-Min Deng[1#], Fang-Fang Dai[1#], Quan Zhou[2*], Yan-Xiang Cheng[1*]

[1]Department of Obstetrics and Gynecology, Renmin Hospital of Wuhan University, Wuhan, Hubei 430060;

[2]Department of Obstetrics and Gynecology, Renmin Hospital of Yichang, Yichang, Hubei 443000;

[#]Contributed equally to this work

[*]***Corresponding author:***

Dr Quan Zhou, Department of Obstetrics and Gynecology, Renmin Hospital of Yichang; Yichang, Hubei 443000; P.R. China;

E-mail: zhouquan8519@163.com

Dr Yan-Xiang Cheng, Department of Obstetrics and Gynecology, Renmin Hospital of Wuhan University; Wuhan, Hubei 430060; P.R. China;

E-mail: rm001050@whu.edu.cn

Supplemental Fig S1. The complete binding site between miRNAs and hsa_circ_0000301. **(A)** hsa-miR-1178-3p & hsa_circ_0000301; **(B)** has-miR-1228-3p & hsa_circ_0000301; **(C)** hsa-miR-767-3p & hsa_circ_0000301.

Supplemental Table S1. Features of the DE-circRNAs in GSE102686 dataset with the cut-off criteria of $|logFC| \geq 2.0$ and adj. $p < 0.05$.

Supplemental Table S2. The target genes of hsa_circ_0000301 in cervical cancer with GEPIA2 and MiRWalk.

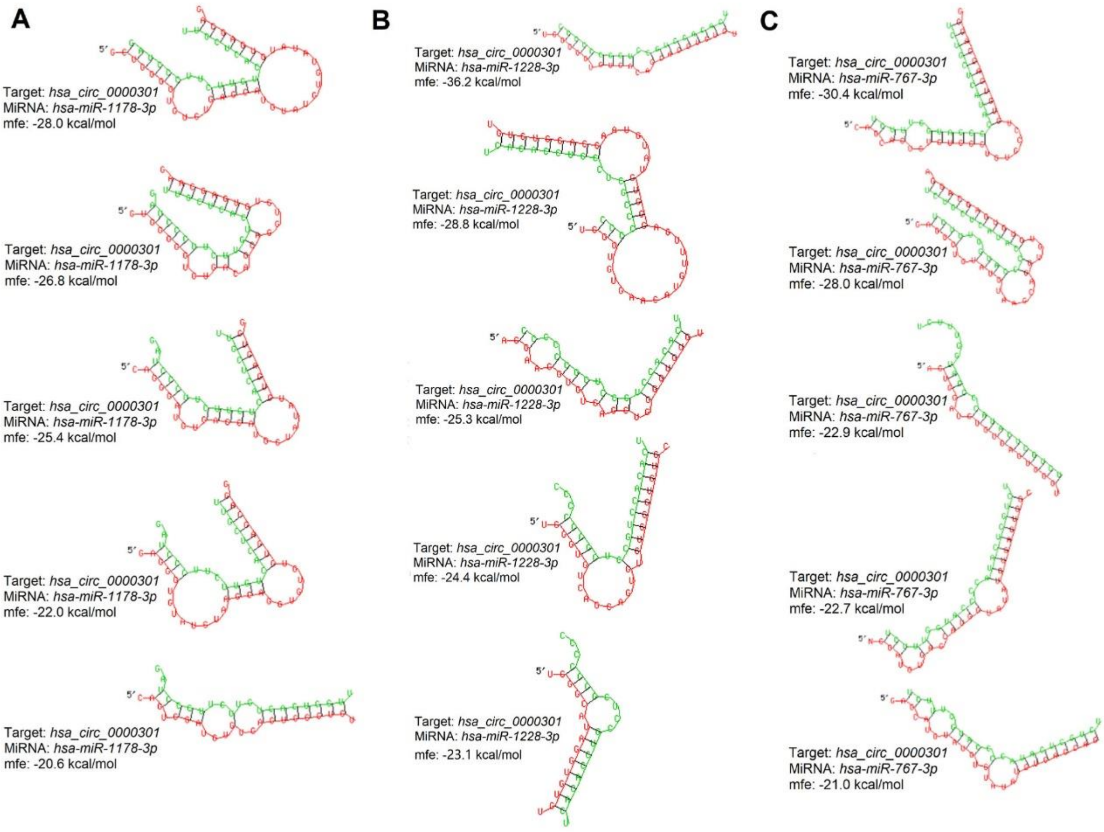
A
Target: hsa_circ_0000301
MiRNA: hsa-miR-1178-3p
mfe: -28.0 kcal/mol
Target: hsa_circ_0000301
MiRNA: hsa-miR-1178-3p
mfe: -26.8 kcal/mol
Target: hsa_circ_0000301
MiRNA: hsa-miR-1178-3p
mfe: -25.4 kcal/mol
Target: hsa_circ_0000301
MiRNA: hsa-miR-1178-3p
mfe: -22.0 kcal/mol
Target: hsa_circ_0000301
MiRNA: hsa-miR-1178-3p
mfe: -20.6 kcal/mol
B
Target: hsa_circ_0000301
MiRNA: hsa-miR-1228-3p
mfe: -36.2 kcal/mol
Target: hsa_circ_0000301
MiRNA: hsa-miR-1228-3p
mfe: -28.8 kcal/mol
Target: hsa_circ_0000301
MiRNA: hsa-miR-1228-3p
mfe: -25.3 kcal/mol
Target: hsa_circ_0000301
MiRNA: hsa-miR-1228-3p
mfe: -24.4 kcal/mol
Target: hsa_circ_0000301
MiRNA: hsa-miR-1228-3p
mfe: -23.1 kcal/mol
C
Target: hsa_circ_0000301
MiRNA: hsa-miR-767-3p
mfe: -30.4 kcal/mol
Target: hsa_circ_0000301
MiRNA: hsa-miR-767-3p
mfe: -28.0 kcal/mol
Target: hsa_circ_0000301
MiRNA: hsa-miR-767-3p
mfe: -22.9 kcal/mol
Target: hsa_circ_0000301
MiRNA: hsa-miR-767-3p
mfe: -22.7 kcal/mol
Target: hsa_circ_0000301
MiRNA: hsa-miR-767-3p
mfe: -21.0 kcal/mol

Fig S1

Supplemental Table S1. Features of the DE-circRNAs in GSE102686 dataset with the cut-off criteria of |logFC| ≥ 2.0 and adj. $p < 0.05$.

Supplemental Table S2. The target genes of hsa_circ_0000301 in cervical cancer with GEPIA2 and MiRWalk.

Table S1. Features of the DE-circRNAs in GSE102686 dataset with the cut-off criteria of |logFC| ≥ 2.0 and adj. $p < 0.05$.

circRNA	logFC	AveExpr	adj.P.Val
hsa_circ_0065898	3.145502	10.63247	2.30E-05
hsa_circ_0070190	2.925457	9.312433	0.000101
hsa_circ_0000077	2.038108	10.09865	0.000692
hsa_circ_0031027	4.278806	11.66952	0.001097
hsa_circ_0000745	-2.76177	4.9717	0.001097
hsa_circ_0043280	2.096494	9.009097	0.001229
hsa_circ_0084927	-2.22328	4.675788	0.007166
hsa_circ_0027821	2.672016	4.411447	0.010788
hsa_circ_0000301	2.059476	6.529432	0.011727
hsa_circ_0020926	2.086247	5.548359	0.020165
hsa_circ_0002762	-2.20839	5.144097	0.031285
hsa_circ_0046290	3.330704	4.952161	0.036329
hsa_circ_0003037	-2.14255	6.105422	0.048003

Note: logFC, log Fold Change; AveExpr, average expression; adj.P.Val, adjusted *p*-value

Table S2. The target genes of hsa_circ_0000301 in cervical cancer with GEPIA2 and MiRWalk.

**Sources of genes**	**Genes**					
TCGA and MiRWalk	KLF6	ZNF385A	IDS	MEOX2	MTR	LONRF2
	ZSCAN18	NR2F1	BCL2	NYNRIN	TFE3	THRA
	EEF2K	GIGYF1	TIMP3	IRF4	VIPR2	JAZF1
	CXCL16	SLC25A42	MEF2D	SBK1	SYNGR1	CSRNP1
	SNX19	GPT2	APBA1	ZIC5	SLC39A13	CD3EAP
	TMX4	TAL1	MEF2C	SLFN13	OAS3	PTGER3
	ANKRD22	WNT7B	MALL	ZNF516	ATF5	
